# Colloidal Titanium Nitride Nanoparticles by Laser Ablation in Solvents for Plasmonic Applications

**DOI:** 10.3390/nano14141214

**Published:** 2024-07-17

**Authors:** Nikolaos Pliatsikas, Stavros Panos, Tamara Odutola, Spyridon Kassavetis, Chrysanthi Papoulia, Ilias Fekas, John Arvanitidis, Dimitris Christofilos, Eleni Pavlidou, Maria Gioti, Panos Patsalas

**Affiliations:** 1 Department of Physics, Aristotle University of Thessaloniki, GR-54124 Thessaloniki, Greece; 2 School of Chemical Engineering and Physics Laboratory, Faculty of Engineering, Aristotle University of Thessaloniki, GR-54124 Thessaloniki, Greece

**Keywords:** pulsed laser ablation in liquids (PLAL), nanosecond laser ablation, titanium nitride nanoparticles, conductive transition metal nitrides, reactive magnetron sputtering, Raman spectroscopy

## Abstract

Titanium nitride (TiN) is a candidate material for several plasmonic applications, and pulsed laser ablation in liquids (PLAL) represents a rapid, scalable, and environmentally friendly approach for the large-scale production of nanomaterials with customized properties. In this work, the nanosecond PLAL process is developed, and we provide a concise understanding of the process parameters, such as the solvent and the laser fluence and pulse wavelength, to the size and structure of the produced TiN nanoparticles (NPs). TiN films of a 0.6 μm thickness developed by direct-current (DC) magnetron sputtering were used as the ablation targets. All laser process parameters lead to the fabrication of spherical NPs, while the laser pulse fluence was used to control the NPs’ size. High laser pulse fluence values result in larger TiN NPs (diameter around 42 nm for 5 mJ and 25 nm for 1 mJ), as measured from scanning electron microscopy (SEM). On the other hand, the wavelength of the laser pulse does not affect the mean size of the TiN NPs (24, 26, and 25 nm for 355, 532, and 1064 nm wavelengths, respectively). However, the wavelength plays a vital role in the quality of the produced TiN NPs. Shorter wavelengths result in NPs with fewer defects, as indicated by Raman spectra and XPS analysis. The solvent type also significantly affects the size of the NPs. In aqueous solutions, strong oxidation of the NPs is evident, while organic solvents such as acetone, carbides, and oxides cover the TiN NPs.

## 1. Introduction

Plasmonics, the interaction of light with metal nanostructures, have been a hot topic over the last years due to their implementation in numerous applications, such as optoelectronics [1,2,3], biophotonics [4,5,6,7], energy conversion [8,9,10,11,12], catalysis, and photochemistry [13,14,15]. While traditional materials like gold (Au) and silver (Ag) have been employed for plasmonics in the visible and near-infrared spectrum, TiN nanoparticles (NPs) can provide localized surface plasmon resonance (LSPR) in the infrared spectral range (700–1100 nm) [16].

Titanium nitride (TiN) is a well-known conductive transition metal nitride due to its unique properties, with refractoriness, high electron mobilities, hardness, and chemical stability among them [17,18,19], and with several industrial applications, such as diffusion barriers, gate electrodes in field-effect transistors and solar cells [20], decorative coating, and in protective and anti-corrosive coatings in cutting tools and machining equipment [19]. In recent years, TiN has been extensively studied for its optical characteristics and, especially, for plasmon resonance, which is a unique phenomenon of light interaction with metallic structures in nanoscale [21,22,23,24].

Colloidal TiN NPs can be fabricated via several methods, such as the direct-current arc discharge method [25], nitridation of TiO_2_ NPs [26], ligand-assisted ammolysis reaction [27], thermal benzene metathesis route [28], and pulsed laser ablation in liquid (PLAL) [23,29,30,31,32]. The latter is a simple, low-cost, easily applicable, environmentally friendly (‘green’), and scalable method for producing NPs of different kinds of materials, such as metals, ceramics, glasses, and composite materials. PLAL can be used for the fabrication of ligand-free NPs that are suitable for biomedical and catalytic applications, unlike the common chemical techniques that require surfactants and additional ligands [33,34]. Ways of fabrication that include chemistry may include multiple preparation steps and employ hazardous products, which can cause extensive contamination of NPs and toxicity issues that deteriorate their performance in specific applications. By adjusting the parameters of the laser process, such as wavelength, repetition time, irradiation time, and pulse energy, but also the type of solvent, the NPs’ characteristics (e.g., morphology, composition, size distribution, concentration, and shape) can be tuned [35]. The optical performance/properties of TiN NPs exhibit notable variations contingent upon the stoichiometry and crystalline quality of the target material. TiN NPs have been fabricated from targets using methods and processes that negatively affect the TiN NPs’ quality. Esmaeilzadeh et al. developed a TiN target by spark plasma sintering (SPS) of TiN powder [29], and Anton A. Popov et al. used a hot-pressed TiN target of 99.5% purity [31] resulting in NPs with irregular shapes and excess of oxygen due to different aqueous and organic solvents. Furthermore, Jingguo Li et al. fabricated directly TiN NPs using nitridation of TiO_2_ powder [36].

The PLAL process is influenced by the nature of the solvent. The composition, structure, or polarity of the solvent’s molecules lead to distinct interactions with NPs, consequently exerting a significant impact on their growth, composition, and stability.

Previous research demonstrated that the solvent-derived species can bind to or react with NPs, inducing alterations in their composition and potentially resulting in an external coating. Moreover, solvent molecules may bind or adsorb to the surface of NPs, impeding their growth and aggregation and contributing to the long-term stability of the colloid. The pivotal role of solvent nature in determining the composition, size/morphology, and stability of NPs is well-documented in numerous scientific papers [37,38,39].

In this work, we develop the target materials (TiN films) using reactive magnetron sputtering to control the NPs’ properties, and we ablate the TiN targets with a nanosecond (ns) laser beam to fabricate high-quality colloidal TiN NPs. The use of ns lasers has not been extensively studied in the literature [29,40], while for the first time, sputtered TiN films were used as target materials, a fact that paves the way for the fabrication of NPs of several other transition metal nitrides. It was verified that the ns pulses of the laser beam enable the diffusion of more energy to the target material, causing refractory materials like TiN to melt and form spherical NPs. Our method leverages the advantages of magnetron sputtering, such as better control over target composition and stoichiometry, resulting in higher-quality TiN NPs. This approach mitigates the issues seen in other methods, such as high defect content and irregular shapes.

## 2. Materials and Methods

### 2.1. Growth of Titanium Nitride Targets

Titanium nitride films with a thickness of 0.6 μm were fabricated by reactive magnetron sputtering at room temperature in a high-vacuum chamber (described in detail in the study by Patsalas et al.) [41], with a base pressure of ~10^−6^ Torr on the silicon samples. The magnetrons operated with independent DC power supplies. The substrate was a horizontal steel plate, positioned 9 cm away from the magnetrons. The substrates were cleaned using an ultrasonic bath for 10 min in acetone and isopropanol, respectively. The sputtering DC power on the 99.95% pure Ti cathode was 2.5 kW; the Ar and N_2_ flows (99.999% pure) were 10 standard cubic centimeters per minute (sccm) and 12 sccm, respectively, resulting in a working pressure of 4.2 mTorr. We used a negative bias voltage of 120 V (which corresponds to an ion energy of 100 eV) on the substrate during the growth.

### 2.2. Synthesis of TiN Nanoparticles

**Figure 1:**
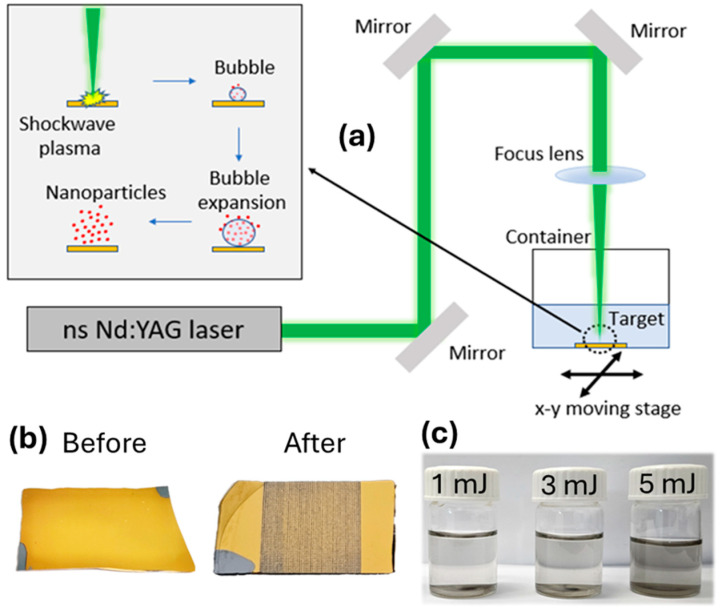
(**a**) Schematic illustration of ablation system, (**b**) photos of the target before and after laser ablation, and (**c**) colloidal solutions of TiN NPs developed by different ns laser pulse energy (1 mJ, 3 mJ, and 5 mJ) at 355 nm.

In this study, laser ablation of TiN films (about 0.6 μm) in liquids was carried out using a nanosecond (ns) lamp-pumped Q-switched Nd:YAG laser (λ = 1064, 532 and 355 nm, 10 Hz repetition rate, 6 ns pulse width, Q-smart 850, Quantel lasers) with a spot size diameter of around 9 mm. The surface of the target was fixed at the bottom of the glass containerfilled with solvents perpendicular to the direction of the incident laser beam (Figure 1a). The height of the liquid above the target was kept at approximately 5 mm to maintain the volume of the solution as low as possible during the laser ablation process. In all experiments, the pulse energy was 1 mJ/pulse, 3 mJ/pulse, and 5 mJ/pulse (RMS stability ~5% RMS (5 h)), as measured by a pyroelectric detector (QE25LP-S-MB-D0, Gentec, Québec City, QC, Canada) to maintain the process stability and prevent ripples and splashing on the surface of the solvent. The laser beam was focused by a plano-convex lens (LA4874-UV, LA4874-YAG (532/1064 nm), Thorlabs, Newton, NJ, USA) with a focal length of f = 200 mm on the surface of the target and a full-focused spot around 50 μm. In the target surface, a near Gaussian (TEM_00_) mode spatial beam profile with a radius (1/e^2^) of (w_o_ = 50 μm) was obtained. The lens-target distance was slightly less than the focusing length of the objective. This happened to ensure the best performance of the laser ablation procedure due to the refraction of the laser beam according to Snell’s law. The glass containerwas up to an x-y translational stage (8MT177-100—Motorized Stage by Standa, Vilnius, Lithuania) that moved along the x-y axis to scan the TiN target and was controlled via a computer interface (Figure 1a). The scanning speed of 2 mm/s was used to prevent pulses from overlapping, which can cause excessive heating of the target materials, and ensure uniform ablation. The pattern of scanning was a back-and-forth movement with a 0.2 mm step. Ten overscans per sample were conducted, producing around 1.5 ± 0.1 mg of NPs per sample. The development was conducted in two different solvents, namely water and acetone. No surfactant agents were used for the stabilization of the colloid. Throughout the ablation process, a considerable portion of volatile acetone solvent evaporated. In cases of solvent evaporation, the glass containerwas refilled, ensuring the volume (and liquid height) of acetone remained at its initial level throughout the process. TiN colloids produced via laser ablation, whether in water or acetone, demonstrated exceptional stability, showing small signs of aggregation or precipitation, even after being stored under ambient conditions for several weeks.

After synthesis, the solution was collected directly by a syringe from the glass vessel, allocated into glass vessels, and examined without any additional post-processing treatment.

### 2.3. Characterization

Spectroscopic ellipsometry (SE) spectra were obtained with a Jobin–Yvon Horiba phase modulated ellipsometer (Horiba JobinYvon, UVISEL, Europe Research Center—Palaiseau, France), at an angle of incidence 70.48°, in the energy region 1.5–5.5 eV, and with a step of 20 meV. The SE experimental data were fitted to model-generated data using a combined Drude–Lorentz model (D2L), taking into consideration all the fitting parameters of the applied model according to Patsalas et al. [18,19].

The concentration of NPs was calculated by measuring the weight loss of the targets before and after the synthesis using an analytical scale with a resolution of 0.1 mg, and the difference was the mass of the material in the solvent. By providing a specific volume of solvents in a glass container, we can calculate the concentration.

The surface chemical states of the produced NPs were analyzed by Raman spectroscopy. Raman measurements were conducted with a micro-Raman Horiba LabRAM HR spectrometer equipped with a Peltier-cooled CCD detector by Horiba, Kyoto, Japan. A 514 nm excitation laser line was focused, with a 100× objective and a power of ~7.5 mW on the sample at a ~1 μm spot size.

A scanning electron microscopy (SEM) technique was used for the morphology characterization and chemical composition of the samples, using a JEOL FESEM JSM 7610FPlus model (JEOL, Tokyo, Japan), equipped with an energy-dispersive X-ray spectroscopy, AZTEC (EDS) analyzer. The operating conditions were 15 kV accelerating voltage, 105 nA probe current, and 60 s counting time.

The crystal structure analysis was performed with X-ray diffraction (XRD) using a Rigaku Ultima + diffractometer (40 kV, 30 mA, CuKα radiation) in Bragg–Brentano geometry (Rigaku, Tokyo, Japan).

The Raman analysis was complemented by X-ray photoelectron spectroscopy (XPS). Photoelectron spectra were obtained using a Kratos AXIS Ultra DLD UHV system (base pressure ~10^−9^ Torr) by Kratos Analytical, Manchester, UK, featuring a monochromated aluminum Ka X-ray source (hv = 1486.6 eV), a hemispherical sector analyzer, and a multichannel detector. A 20 eV pass energy is utilized, resulting in a full width at half maximum (FWHM) for the Ag-*3d*_5/2_ peak of less than 500 meV, with a step size of 0.1 eV. XPS data were analyzed using Kratos Vision software with built-in relative sensitivity factors.

## 3. Results and Discussion

### 3.1. Characterization of TiN Films

**Figure 2:**
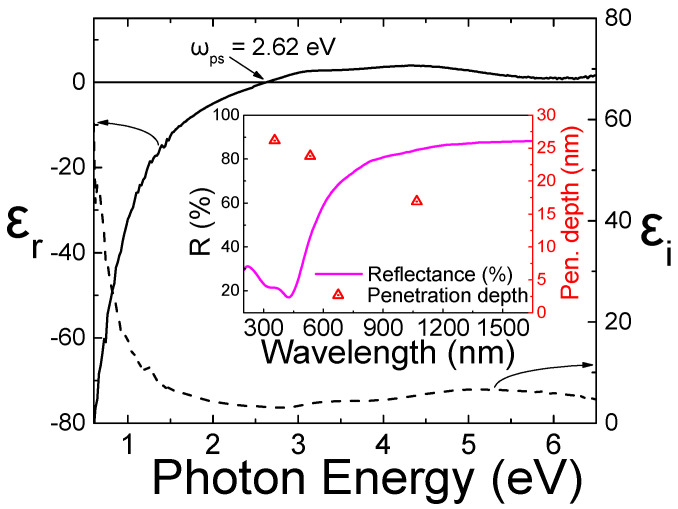
SE spectra of the fabricated TiN film deposited by UBRMS. Inset plots: reflectance spectrum of the TiN film on top of silicon substrate and the calculated optical penetration depth of the laser beam in the TiN films.

Figure 2 shows the real (ε_r_) and the imaginary (ε_i_) part of the TiN (0.6 μm thickness) pseudodielectric function deposited on Si substrates. The deposition was carried out with the nitrogen (N_2_) flow rate set to 12 sccm and controlled by adjusting the nitrogen mass flow controller. The TiN film exhibits excellent metallic behavior (negative permittivity, ε_r_ < 0) in the visible to near-infrared spectral regions for photon energy < 2.7 eV. This means that the TiN film is potentially plasmonic in the respective spectral region (this also can be proved in the inset plot of Figure 2). Also, the value of the apparent plasma energy (ω_ps_) can be used as a criterion for the stoichiometry of TiN_x_ films according to the equation 2.36 < ω_ps_ < 2.6 ⇔ 1.07 < [N]/[Ti] < 1.01, as described in detail by Patsalas et al. [41]. In this case, ω_ps_ = 2.62 eV, indicating that the TiN film is stoichiometric.

In the inset plots of Figure 2, we present the reflectance spectra for 0.6 μm TiN films deposited on a Si substrate under previously described conditions. The reflectance, denoted as R, initially reaches a minimum value of 16% at a wavelength of approximately 425 nm. Subsequently, it sharply rises, close to 90%, as it extends towards near-infrared wavelengths. The observed reflectance characteristics closely mirror those of Au films, providing validation for the highly plasmonic and metallic nature of our TiN films [18,42]. The reflectance minimum in TiN occurs on the high-energy side, where strong interband transitions dominate, while the reflectance maximum in the longer wavelength region is a result of the high metallicity of TiN. The potentially plasmonic quality of our TiN films is further evidenced by their gold-like color, as depicted in Figure 1b.

In Figure S3, we show the X-ray diffraction (XRD) patterns of TiN films sputtered on silicon substrate surfaces. All TiN films exhibit a consistent crystalline structure, characterized by three peaks at approximately 36.2°, 42.5°, and 61.6°, corresponding to the (111), (200), and (220) orientations, respectively. The presence of these peaks in XRD suggests the polycrystalline nature of our room-temperature sputtered TiN films. The grain size (D) estimated using Scherrer’s formula in the (200) orientation is D = 9.77 nm, and it is comparable to that reported for reactive sputtered TiN films elsewhere [43,44].

### 3.2. Fabrication and Characterization of Colloidal TiN Nanoparticles

During the PLAL process, a laser beam of a wavelength in the absorption spectral area of TiN and with a laser fluence that surpasses the material’s ablation threshold generates a plasma plume of ablated species (neutral atoms or ions) at the point of incidence on the material’s surface. In the context of ns pulse laser ablation, the energy delivery to the TiN material is gradual; the plasma plume originates from the vaporization of the material melt pool. Specifically, the material undergoes ablation through a process of melting and vaporization, in contrast with ultrashort pulse laser ablation where the ablation occurs through sublimation (direct transition from solid to vapor) [45]. The elevated temperature within the plume induces the ionization and vaporization of the liquid at the interface between the plume and the liquid, transforming it into liquid vapor as well as atomic and molecular hydrogen, oxygen, or carbon (depending on the solvent).

**Figure 3:**
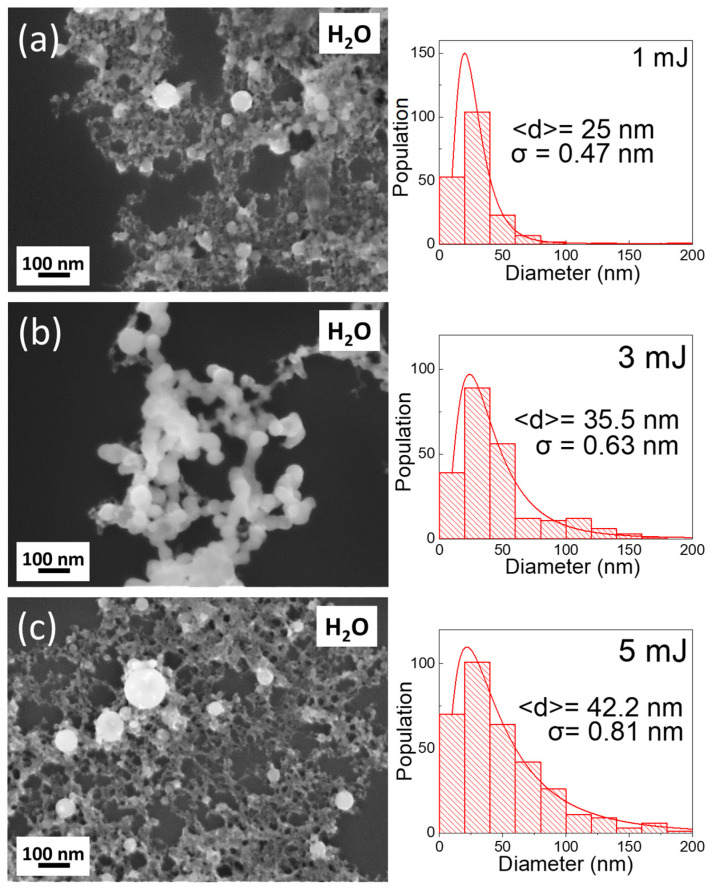
SEM images and corresponding size-distribution histograms of the NPs developed with (**a**) 1 mJ, (**b**) 3 mJ, and (**c**) 5 mJ, respectively, at 1064 nm laser wavelength.

First, the effect of the laser pulse energy (E) in the formation of TiN NPs was examined. SEM characterization of the TiN NPs showed that the developed NPs are perfectly spherical for E ranging from 1 mJ to 5 mJ. The size-distribution histograms for the NPs produced by a 1064 nm beam at 1, 3, and 5 mJ are shown in Figure 3. ImageJ software (Version 1.54h) was used to analyze the SEM images and conclude the size distribution of the TiN NPs. Five SEM images of each sample were analyzed for better precision. These histograms are effectively characterized by log-normal functions, featuring median diameters of <d> = 25.0, 35.5, and 42.2 nm along with standard deviations of σ = 0.47, 0.63, and 0.81, respectively. These lower-energy laser pulses result in smaller TiN NPs and narrower size distributions compared to higher-energy laser pulses (see Figure 3). Similar results were observed for the cases of irradiation at 355 nm and 532 nm.

Concerning laser parameters, it is established that various factors, such as the penetration depth of the laser beam, self-absorption, and ablation efficiency, are intricately linked to the laser wavelength, significantly influencing outcomes. Specifically, the absorption efficiency of the incident laser energy dictates the size and/or concentration of the resulting NPs. As shown in Figure 2, TiN films exhibit maximum absorption at wavelengths between 300 and 450 nm. Consequently, with ns pulse duration, plasma heating is significantly greater at 355 nm compared to 532 nm, and especially to 1064 nm. To further study the effect of ns laser pulse wavelength on the size of the TiN NPs, the same experimental conditions were maintained (pulse energy—1 mJ, liquid—water, focusing lens—200 mm) while selecting three different ns laser pulse wavelengths (355 nm, 532 nm, and 1064 nm).

As observed from the SEM images, the developed NPs remain spherical across all cases. The size-distribution histograms for the TiN NPs developed at 355 nm, 532 nm, and 1064 nm are shown in Figure 3a–c. These histograms are effectively characterized by log-normal functions, featuring median diameters of <d> = 24.2, 26.6, and 25.0 nm, along with standard deviations of σ = 0.50, 0.48, and 0.47, respectively. In this case, the median diameter is approximately the same, while bigger TiN NPs were developed by the ns laser pulse of a 355 nm wavelength. Meanwhile, in the case of the ns laser pulse of 1064 nm wavelength, the size distribution of the TiN NPs is slightly narrower.

The above result can be explained by considering that, in the nanosecond pulses (*τ_L_*), the thermal penetration depth exceeds the optical penetration depth. On that occasion, the pulse duration exceeds the lattice heating time, resulting in the equilibrium of the lattice and electron, and the thermal penetration length (*l_t_*) is a function of the thermal diffusivity (D_T_) of the target material given by the following equation [46], *l_t_* ≈ √(D_T_ · *τ_L_*) (1). The optical penetration (*l_a_*) of the laser on the target material is a function of the attenuation coefficient (a) given by the equation [46] *l_a_ =* 1/a (2). In the inset plots in Figure 2, the optical penetration depth in TiN films is calculated to be 26.2 nm, 23.8 nm, and 16.9 nm for the laser pulse wavelengths of 355 nm, 532 nm, and 1064 nm, respectively. On the other hand, the thermal penetration length is around 70 nm [47]. Thus, the actual melting pool from which the TiN NPs are generated depends on the thermal heat-penetration depth in combination with the laser beam power and not on the ns laser pulse wavelength. In fact, according to Nolte et al. [48], only for ultra-short laser pulses < 10^−9^ s (picosecond or femtosecond regime), the optical penetration length can play a significant role in the ablation process and the NPs’ characteristics.

**Figure 4:**
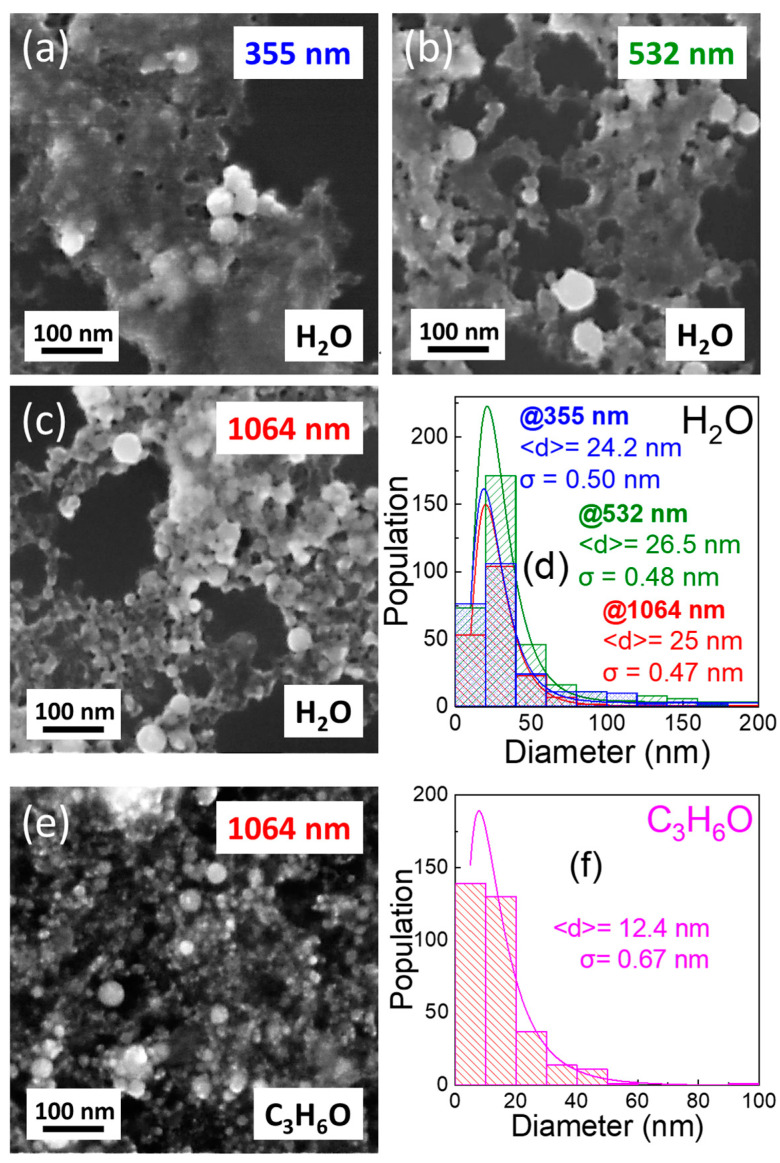
SEM images of the NPs developed in water for (**a**) 355 nm, (**b**) 532 nm, and (**c**) 1064 nm pulse laser wavelengths, respectively, and (**d**) the corresponding size-distribution histograms. (**e**) SEM image of the NPs developed in acetone for 1064 nm pulse laser wavelength and (**f**) the corresponding size-distribution histograms. For all SEM images, the pulse energy is 1 mJ.

For the effect of the solvent, the TiN NPs developed in acetone were smaller in size (<d> = 12.4 and σ = 0.67) compared to those developed in water, underscoring the considerable influence of the solvent nature on NPs formation and growth, as depicted in Figure 4e,f). Previous research has elucidated the pivotal role of carbon, derived from organic solvents like acetone, in the nucleation and growth of NPs [49]. In contrast, water promotes oxidation of the ejected material by molecular oxygen. Carbon species originating from acetone serve to shield the surfaces of particles after the pyrolysis of organic solvents, thereby impeding further growth and agglomeration. Consequently, TiN-NPs developed in the organic solvent demonstrate a smaller average size than those synthesized in water under identical processing conditions [39,50].

Analysis of the chemical composition of TiN NPs was conducted, using an energy-dispersive X-ray spectroscopy (SEM/EDS) technique. An illustrative EDS spectrum, depicting the composition of the PLAL-developed TiN NPs, is shown in Figure S3. The EDS spectrum reveals the presence of oxygen, nitrogen, silicon, and titanium. The appearance of a silicon peak in the spectrum is attributed to the background signal, originating from the Si wafer, which is used as a mount/base for the TiN NPs. The observation of Ti confirms the existence of TiN NPs but not the purity/quality of them.

**Figure 5:**
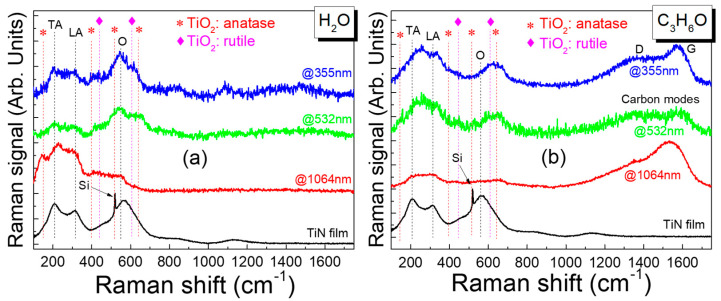
Micro-Raman spectra of TiN NPs produced in (**a**) water and (**b**) acetone with different ablation wavelengths: 355 (blue line), 532 nm (green line), 1064 nm (red line), and bulk TiN reference (black line).

The quality of TiN NPs in the colloidal solutions was studied by micro-Raman characterization. Figure 5 shows the Raman spectra of TiN NPs produced in water with 1 mJ pulse laser energy at 355 nm, 532 nm, and 1064 nm ablation wavelengths. It is known that, in the Raman spectra of TiN, the doublet at low frequencies (210–220 cm^−1^ for transverse acoustic (TA) phonons and 300–320 cm^−1^ for longitudinal acoustic (LA) phonons) is related to the presence of nitrogen vacancies, while the optical band located at 520–580 cm^−1^ (originating from both the transverse optical (TO) and the longitudinal optical (LO) phonons) is attributed to titanium vacancies [51,52,53,54]. All our samples show the above characteristic peaks; thus, both nitrogen and titanium vacancies are present. Additionally, the spectral features associated with the presence of titanium dioxide (TiO_2_) in rutile and anatase structures appear in the Raman spectra of the NPs. The presence of TiO_2_ is possibly due to either the formation of an oxidized shell around the NPs or the incorporation of a large number of oxygen atoms in the TiN matrix (TiN films).

**Figure 6:**
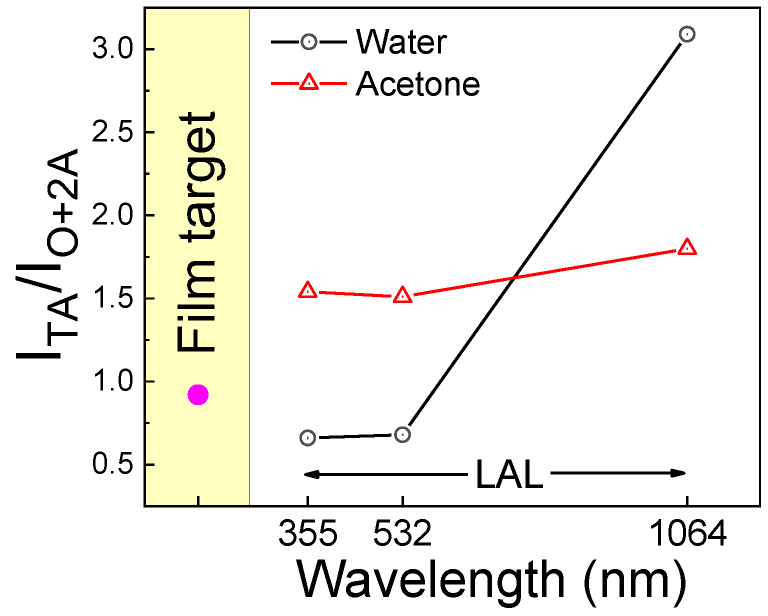
Relative intensities of the acoustic transverse Raman peak over the optical band for TiN NPs ablated at 355, 532, and 1064 nm in water (black line) and acetone (red line), as well as that for the thick film after deposition (pink point). The intensities were extracted from the highest point of each peak after the subtraction of the background.

Spengler et al. [54] associated the intensity ratio of the first-order acoustic modes to the broad envelope, encompassing the O and 2A modes with the density of point defects that disrupt the local Oh symmetry for B1-TiN_x_ [53]. From Figure 5a, it is apparent that the colloidal NPs fabricated with an ns laser pulse of 355 nm and 532 nm wavelengths have fewer point defects than those fabricated with an ns laser pulse of 1064 nm. This is further visualized in Figure 6, where the relative intensities of the acoustic transverse Raman peaks over the optical bands for TiN NPs and the film are illustrated (black line). Surprisingly, it also appears that the NPs produced through 532 nm and 355 nm have fewer defects than the TiN film due to the high temperature during the PLAL by the ns laser pulse development of the TiN NPs. It is well known that high-temperature anneals out the defects in TiN [52,55,56].

In Figure 5b, micro-Raman spectra of TiN NPs produced in acetone at 355 (blue line), 532 nm (green line), and 1064 nm (red line) are deployed. The doublet peak at low frequencies (210 cm^−1^ and 310 cm^−1^) corresponds to the transverse acoustic (TA) and longitudinal acoustic (LA) photons, respectively, and the optical band located at 520–580 cm^−1^ associated with optical phonons is observed. Spectral features associated with the presence of TiO_2_ appear in the Raman spectra of the NPs produced in acetone, similar to the case when water is used as the solvent. The appearance of TiO_2_ peaks suggests partial oxidation of the TiN NPs due to the acetone environment. Additionally, spectral features at 1340 cm^−1^ and 1575 cm^−1^ are clearly observed, corresponding to the D and G carbon modes, respectively. The lineshapes and intensity ratio of the D/G peaks reflect the presence of amorphous carbon on the surface of the TiN NPs [57,58]. This carbon contamination occurs during the PLAL process in acetone, affecting the overall quality and properties of the TiN NPs. The presence of these carbon modes suggests that the acetone environment facilitates the deposition of carbonaceous material onto the NPs.

The relative intensity of the TA over the O mode indicates that irradiation with wavelengths at 355 nm and 532 nm results in NPs with fewer point defects compared to 1064 nm. Although this is similar to the results in water, the intensity is much lower (see Figure 6, red line, 1064 nm). This lower intensity is likely due to the ease with which impurities are incorporated into NPs because of the carbon shell surrounding them. The presence of a carbon shell can also explain the interesting phenomenon of fewer point defects at 355 nm and 532 nm in the water solution. The carbon shell around the NPs acts as a barrier to defects, thereby inhibiting their relaxation through thermal processes when using an ns laser. Thus, in acetone, the carbon shell forms a passivation layer that both prevents defects from escaping the NPs (355 nm and 532 nm) and prevents impurities from entering the NPs (1064 nm). We can conclude that NPs follow the same motif; laser ablation using the 355 nm and 532 nm beam wavelengths results in better crystalline quality NPs compared to those developed by the ns laser pulse with a 1064 nm beam wavelength. In all cases, the formation of impurities is independent of the initial state of the TiN film target, since all film targets were identical. Consequently, the presence of impurities is attributed to the PLAL process and the solvent used, rather than to the sputtered TiN film itself.

**Figure 7:**
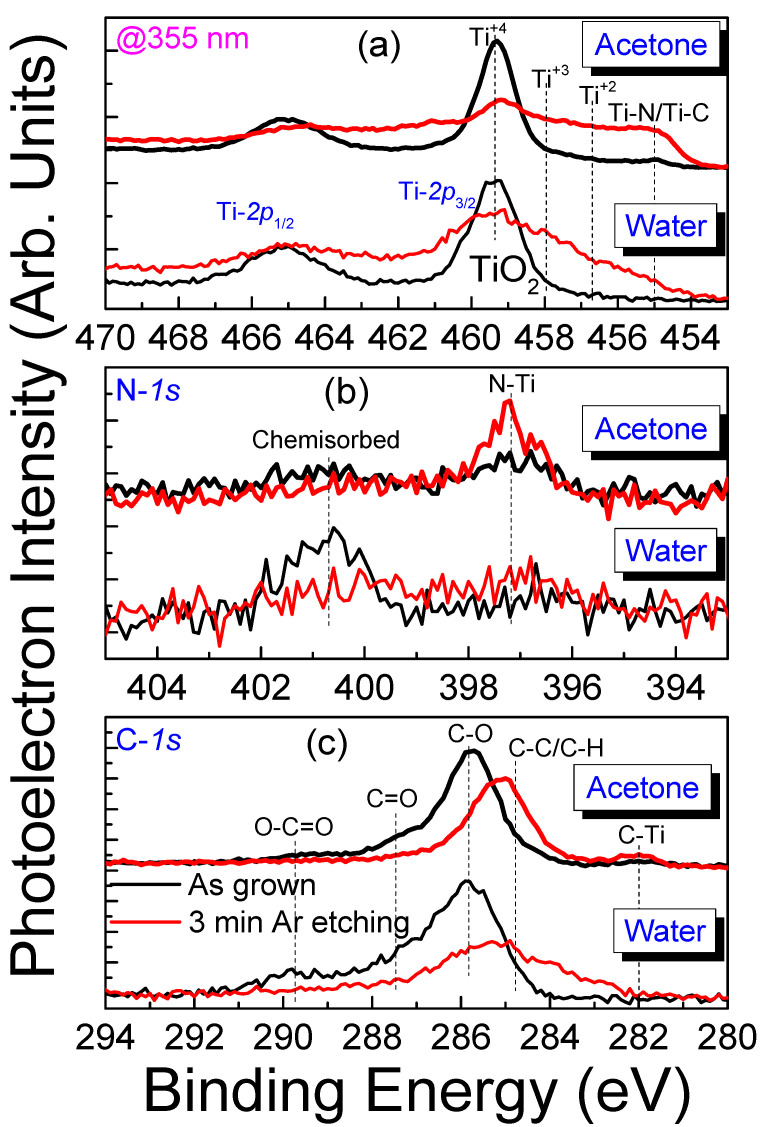
Core-level spectra of (**a**) Ti-*2p*, (**b**) N-*1s*, and (**c**) C-*1s* before and after Ar gun etching of the TiN NPs developed in water and acetone at 355 nm wavelength. The black line corresponds to the NPs as produced while the red line corresponds after 3 min etching.

For a better understanding of the surface chemical composition of the developed TiN NPs, we used XPS analysis. Figure 7 presents the core-level spectra of Ti-*2p*, N-*1s*, and C-*1s* for TiN NPs developed in water and acetone before and after Ar gun etching. In Figure 7a, the Ti-*2p* spectra before etching of the TiN NPs developed in water and acetone are dominated by the double symmetric peak Ti-*2p*_3/2_ and Ti-*2p*_1/2_ of TiO_2_ at 459.3 and 465 eV, respectively [59,60,61]. This means that the TiN NPs, either in the case of water or in the case of acetone, are surface oxidized. In fact, due to the relative intensity of the Ti-*2p*_3/2_ peak to the Ti-*2p*_1/2_ peak of TiO_2_ being more than two, the NPs are mainly dominated by TiO_2_ [59]. In the case of the TiN NPs developed in acetone, a peak at 455 nm corresponding to the TiN structure appears. The intensity of this peak increases greatly after etching [59,60,61,62,63]. This can be explained by the existence of core-shell NPs, with TiN as a core and TiC as a shell, which is in agreement with the micro-Raman results that show the D and G carbon peaks at 1340 and 1575 cm^−1^ (see Figure 5b). In water, the Ti-*2p*_3/2_ peak of the oxide also decreases after etching, and the Ti-*2p*_3/2_ peak of TiN at 455 nm appears after 3 min of etching [64].

The analysis of the N-*1s* spectrum (Figure 7b) shows the peak at 397.1 nm of the N-Ti bond more strongly in the case of TiN NPs developed in acetone, while in the case of the TiN NPs developed in water, a peak appears at around 401 nm, which can be attributed either to adsorbed nitrogen atoms (N_2_) or to the NO_x_ bonds [65]. In both cases, with water and acetone as solvents, the N-Ti peak increases after Ar etching, which is consistent with the Ti-N peak increase at 455 eV in the Ti-*2p*_3/2_ peak. The C-*1s* spectrum (Figure 7c) shows similar behavior in both TiN NPs developed in water and acetone. It shows the characteristic peaks of C-C, C-O, C=O, and C-Ti bonds appearing at 285.1 eV, 287.6 eV, and 289.7 eV, respectively [61,66]. In the case of acetone, there is another peak at 281 eV, which in the literature is attributed to inorganic carbides. Therefore, it can be attributed to the titanium carbide (TiC) bond [67], confirming what was reported in the analysis of the Ti-*2p* spectrum. Therefore, from the overall XPS analysis, the TiN NPs contain amorphous TiO_2_ both in water and acetone, while in acetone, Ti-C bonds also appear, indicating the presence of carbides.

## 4. Conclusions

We present a comprehensive analysis of the development and characterization of colloidal TiN NPs produced via pulsed laser ablation in liquids (PLAL) using nanosecond laser pulses of various wavelengths and energies. Our findings demonstrate the effectiveness of the PLAL method for producing high-quality TiN NPs with controlled sizes and properties. The development of TiN NPs using nanosecond laser pulses is particularly noteworthy, as it has not been extensively studied. Our work establishes nanosecond PLAL as a viable and effective process for producing plasmonic TiN NPs suitable for various applications.

We found that laser fluence plays a significant role in the size of the TiN NPs, which is crucial for tailoring the optical and electronic properties of the NPs for specific plasmonic applications. Higher PLAL fluence values lead to larger TiN NPs, with 5 mJ producing NPs with a mean diameter of around 42 nm and a lower fluence of 1 mJ resulted in smaller NPs with a mean diameter of approximately 25 nm. Additionally, we observed that, while the ns laser pulse wavelength did not significantly affect the mean size of the NPs, it notably influenced the crystalline quality. Specifically, shorter wavelengths resulted in TiN NPs with fewer defects, as evidenced by Raman analysis. The solvent used in the PLAL process also significantly impacts the size and quality of the TiN NPs. In aqueous solutions, the NPs exhibited strong oxidation, while in organic solvents such as acetone, the NPs were covered with carbides and oxides.

Overall, the herein-presented research results highlight the potential of nanosecond PLAL as a scalable, environmentally friendly method compared to chemical synthesis for producing TiN NPs with tunable sizes, particularly when using a higher repetition rate laser system (e.g., >10 kHz). The control of the TiN NP size through laser fluence and the improvement of their crystalline quality using shorter wavelengths denotes or emphasizes the versatility of the PLAL process for the production of TiN NPs for advanced plasmonic applications, including optoelectronics, biophotonics, and energy-conversion ones.

## Data Availability

Data are contained within the article.

## Supplementary Materials

The following supporting information can be downloaded at https://www.mdpi.com/article/10.3390/nano14141214/s1: Figure S1: XRD pattern from the TiN film on Si; Figure S2: NPs size compared to laser pulse energy used in ablation process at 1064 nm wavelength. The inset image presents the actual colloidal NPs produced in the marginal pulse energies; Figure S3: Energy dispersive X-ray spectroscopy (EDS) spectrum of produced TiN NPs.
