# Supplementary material for: Colloidal Titanium Nitride Nanoparticles by Laser Ablation in Solvents for Plasmonic Applications
Source: Nanomaterials (Basel). 2024 Jul 17;14(14):1214. doi: 10.3390/nano14141214 (PMC11279895; doi:10.3390/nano14141214)
Supplement: Supplementary file 1 [file nanomaterials-14-01214-s001.zip › nanomaterials-3068837-supplementary.pdf]

Supplementary material

Colloidal titanium nitride nanoparticles by Laser ablation in solvents for plasmonic applications

Nikolaos Pliatsikas [1,*], Stavros Panos [1], Tamara Odutola [1], Spyridon Kassavetis [1], Chrysanthi Papoulia [1], Ilias Fekas [1], John Arvanitidis [1], Dimitris Christofilos [2], Eleni Pavlidou [1], Maria Gioti [1] and Panos Patsalas [1]

[1] Department of Physics, Aristotle University of Thessaloniki, GR-54124 Thessaloniki, Greece; stpanos@physics.auth.gr (S.P.); tontouto@physics.auth.gr (T.O.); skasa@physics.auth.gr (S.K.); cpapouli@physics.auth.gr (C.P.); ifekas@physics.auth.gr (I.F.); jarvan@physics.auth.gr (J.A.); elpavlid@auth.gr (E.P.); mgiot@physics.auth.gr (M.G.); ppats@physics.auth.gr (P.P.)

[2] School of Chemical Engineering and Physics Laboratory, Faculty of Engineering, Aristotle University of Thessaloniki, GR-54124 Thessaloniki, Greece; christop@cheng.auth.gr

* Correspondence: nipliats@physics.auth.gr

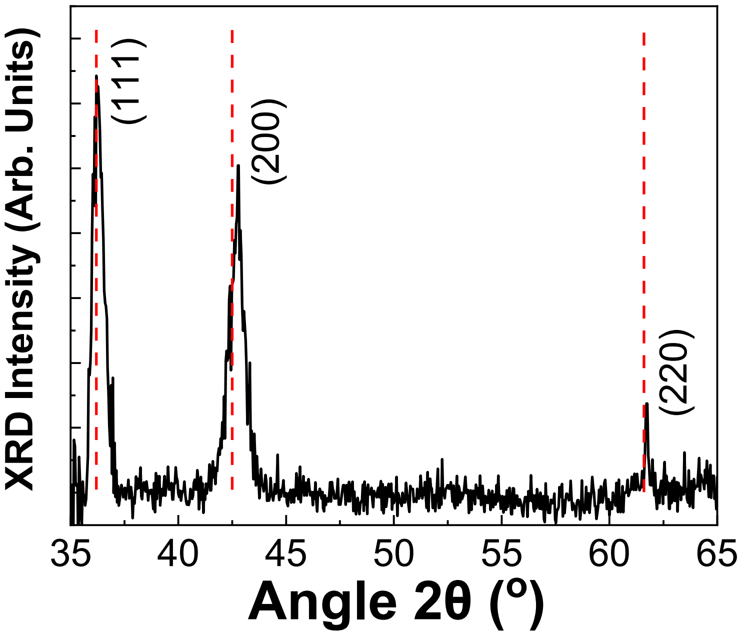

Figure S1: XRD pattern from the TiN film on Si.

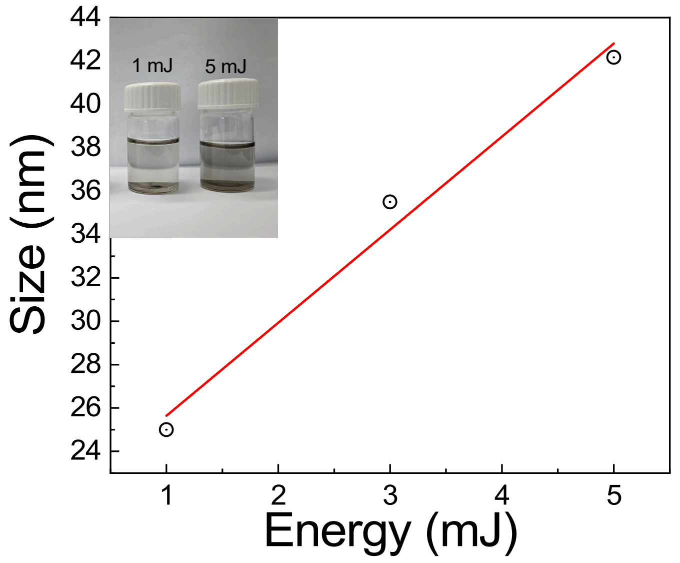

Figure S2: NPs sizes compared to laser pulse energy used in the ablation process at 1064 nm wavelength. The inset image presents the actual colloidal NPs produced by the marginal pulse energies.

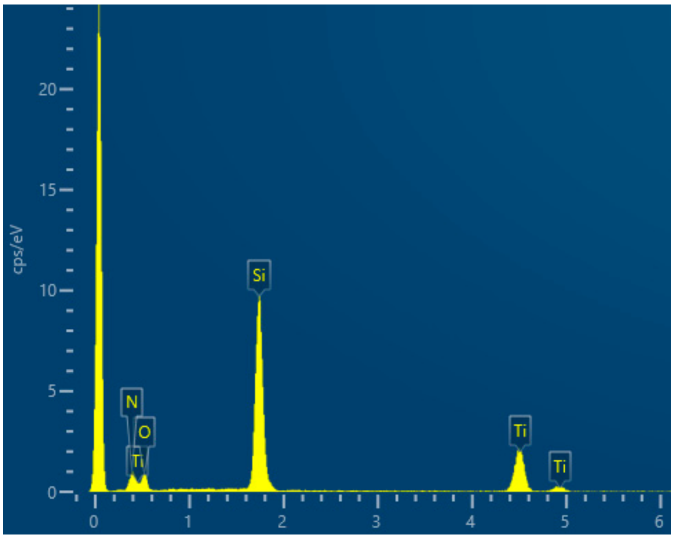

Figure S3: Energy-dispersive X-ray spectroscopy (EDS) spectrum of produced TiN NPs
